# Novel Hydrogel Material with Tailored Internal Architecture Modified by “Bio” Amphiphilic Components—Design and Analysis by a Physico-Chemical Approach

**DOI:** 10.3390/gels8020115

**Published:** 2022-02-13

**Authors:** Richard Heger, Martin Kadlec, Monika Trudicova, Natalia Zinkovska, Jan Hajzler, Miloslav Pekar, Jiri Smilek

**Affiliations:** 1 Materials Research Center, Faculty of Chemistry, Brno University of Technology, Purkynova 118, 61200 Brno, Czech Republic; martin.kadlec@vut.cz (M.K.); xctrudicova@fch.vut.cz (M.T.); xczinkovska@fch.vut.cz (N.Z.); 2 Institute of Physical and Applied Chemistry, Faculty of Chemistry, Brno University of Technology, Purkynova 118, 61200 Brno, Czech Republic; 3 Institute of Materials Science, Faculty of Chemistry, Brno University of Technology, Purkynova 118, 61200 Brno, Czech Republic; xchajzler@fch.vut.cz

**Keywords:** lecithin, hydrogel, rheology, scanning electron microscopy, drying and swelling, extracellular matrix, mesh size

## Abstract

Nowadays, hydrogels are found in many applications ranging from the industrial to the biological (e.g., tissue engineering, drug delivery systems, cosmetics, water treatment, and many more). According to the specific needs of individual applications, it is necessary to be able to modify the properties of hydrogel materials, particularly the transport and mechanical properties related to their structure, which are crucial for the potential use of the hydrogels in modern material engineering. Therefore, the possibility of preparing hydrogel materials with tunable properties is a very real topic and is still being researched. A simple way to modify these properties is to alter the internal structure by adding another component. The addition of natural substances is convenient due to their biocompatibility and the possibility of biodegradation. Therefore, this work focused on hydrogels modified by a substance that is naturally found in the tissues of our body, namely lecithin. Hydrogels were prepared by different types of crosslinking (physical, ionic, and chemical). Their mechanical properties were monitored and these investigations were supplemented by drying and rehydration measurements, and supported by the morphological characterization of xerogels. With the addition of natural lecithin, it is possible to modify crucial properties of hydrogels such as porosity and mechanical properties, which will play a role in the final applications.

## 1. Introduction

Hydrogels are hydrophilic polymers with a three-dimensional network structure that have the ability to absorb a large volume of water due to the presence of hydrophilic moieties, which makes them particularly suitable materials for biomedical applications (e.g., scaffolds) [1]. Selecting the pertinent components for the fabrication of the final hydrogel allows for a functional and applicable material with unique properties (e.g., porosity, biocompatibility, biodegradability) to be obtained. This exact customizable functionality makes these materials appropriate and desirable for a wide range of application areas (tissue engineering, pharmacy, water treatment, material engineering, etc.).

An equally important property of hydrogels is their ability to simulate and mimic biological systems such as the extracellular matrix (ECM), which is, in fact, a structural support network composed of diverse proteins, sugars, and other components. ECM regulates cellular processes including survival, growth, proliferation, migration, and differentiation [2]. Engineering a tailored in vitro environment mimicking the organized structure of ECM is a huge challenge and a desired goal. Since the scaffolds must offer relevant properties sufficient for cellular function, hydrogels have an advantage as potential materials due to their tunable physico-chemical (electrical charge and pore size) and mechanical (stiffness, tensile strength) properties [3]. The majority of hydrogels are also biocompatible, for example, naturally derived polymers such as agarose, alginate, chitosan, collagen, fibrin, gelatin, hyaluronic acid, and dextran as well as biocompatible synthetic gels based on poly(ethylene glycol) (PEG), poly(vinyl alcohol) (PVA), and poly(hydroxyethyl methacrylate) (PHEMA) [4].

Since the 3D network structure of hydrogels is mainly responsible for their mechanical properties and porous microstructure, one of the possibilities of how to modify, upgrade, or tailor properties of hydrogels is to incorporate hydrophobic or micellar domains into the gel structure [5].

Pure hydrophobic association (HA) hydrogels refer to physically crosslinked hydrogels formed by hydrophobic interactions, which account for 5–20% of the total amount of polymer. The bulk of hydrophobic association hydrogels are produced by micellar copolymerization [6]. For instance, Tuncaboylu et al. attempted to improve the low mechanical strength of self-healing hydrogels by creating hybrid hydrogels with strong hydrophobic interactions between hydrophilic polymers mediated by the large hydrophobic moiety of a physical crosslinker (stearyl methacrylate) [7]. The addition of NaCl to the reaction solution during the copolymerization of large hydrophobes (stearyl methacrylate (C18)) with the hydrophilic monomer acrylamide (AAm) in an aqueous solution of sodium dodecyl sulfate (SDS) led to micellar growth and the solubilization of the large hydrophobes within the SDS micelles. Rheological measurements showed that the hydrophobic associations surrounded by surfactant micelles acted as reversible breakable crosslinks responsible for the rapid self-healing of the hydrogels [7].

An alternative approach to enhance the toughness of the hydrogel network is to introduce particles as additional crosslinking points (e.g., latex particles, nanoparticles) [6]. Latex particles (LPs) that are usually prepared via emulsion polymerization ensure effective energy dissipation and provide hydrogels with higher mechanical properties. Gu et al. [8] proposed a method that encompassed the adsorption of the hydrophobic alkyl chains of hydrophobic monomers on the surface of the latex microspheres and their subsequent stabilization in the presence of surfactants, thus forming hydrophobic association centers as the first physical crosslinking points. Moreover, anionic sulfate radicals (originating from the dissociation of the persulfate) were attracted toward the cationic chains of latex microspheres (obtained via surfactant-free emulsion copolymerization of styrene with a vinylidene comonomer bearing a cationic side group) and formed secondary physical crosslinking centers. The incorporation of cationic latex microspheres led to an improvement in the tensile and compression strength of the modified hydrogel compared with pure hydrophobic association hydrogel.

Since inorganic nanoparticles have a high specific surface area, their incorporation into the hydrogel network could also improve its mechanical behavior relating to surface structure and charging [6]. At the same time, the introduction of calcium carbonate nanoparticles [9], hydroxyapatite [10], kaolin [11], and laponite particles [12] could also induce hydrogel adhesion.

On the other hand, the embodiment of polymeric nanoparticles provides the ability to encapsulate both hydrophobic and hydrophilic substances [6]. In addition, Arno et al. investigated how particle morphology (e.g., particle shape, size, and surface) affected the adhesion and mechanical properties of the resultant calcium-alginate hydrogels [13]. The authors demonstrated that 2D platelets substantially improved both the adhesion between hydrogel surfaces and the material’s mechanical strength when blended into the polymeric network compared to their 0D spherical or 1D cylindrical counterparts.

The properties of hydrogels, as mentioned previously, can be adapted not only through the appropriate choice of materials and crosslinking techniques, but also by modifying the internal structure of the gel by using a structure modifier such as lecithin during the preparation process. It should be remembered that lecithin is a typical amphiphilic phospholipid mixture primarily containing distearoylphosphatidylcholine, which possesses good biocompatibility and capability to enhance the bioavailability of co-administered drugs [14]. Lecithin in water systems can self-assembly into array of liquid-crystalline structures depending on the amount of water and temperature. The most likely structures formed under normal working laboratory conditions are lamellar liquid-crystalline structures [15]. Moreover, varying the ratio of lecithin in the multi-component hydrogel system may further improve the applicability and functionality of designed gels. The transport and mechanical properties of materials are given by their internal structure and can be greatly affected by its rearrangement.

Among the different types of lecithin-based systems, the most common platforms in this area are liposomes and microemulsions [16]. Liposomes are an example of soft phospholipid nanoparticles with typical diameters of around 100 nm [17]. Due to their closed vesicular structure, hydrophilic active compounds could be embedded into their internal water compartments, while hydrophobic compounds could be loaded into the bilayer of the liposome. In most cases, lecithin-based liposomal hydrogels are used as carriers; nevertheless, such systems still have certain disadvantages such as a slow and uncontrolled process of drug release [18]. In contrast, lecithin microemulsion-based gels or organogels have some advantages over liposomal hydrogels such as an easier preparation procedure, an absence of organic solvents, and higher storage stability due to the thermodynamic stability of microemulsions [19]. The matrix of lecithin microemulsion-based gels is composed of lecithin, which acts as a surfactant as well as a gelling agent in the presence of a nonpolar organic solvent (external phase) or a polar agent, which is usually water.

Substantial research is focused on modifying the internal structures of hydrogels, however, to the best of our knowledge, there has previously been no systematic study investigating the preparation and targeted modification of the internal structures of biocompatible hydrogels that focused on the use of natural amphiphilic substances and their crucial (e.g., mechanical) application properties.

Thus, this work focuses on the effect of the structure modifier lecithin (as stated before, the lecithin is able to self-organize into liquid-crystalline structures) and its concentration on the resultant mechanical properties of differently crosslinked hydrogels. The results of this work could provide a deeper understanding of the interactions between lecithin and the hydrogel network, and, alternatively, between lecithin and model drugs. Lecithin aggregates in hydrogels can also be viewed as a model of phospholipid structures (like cell membranes) occurring in real tissues, and thus as a model of their potential impact on the rheological or transport properties of the extracellular matrix.

## 2. Results and Discussion

On the basis of the prior experience of our team and in an attempt to investigate the effect of different crosslinking strategies on the final properties of hydrogels, the following materials were selected: agarose as a physically crosslinked hydrogel, alginate crosslinked by polyvalent ions as an ionically crosslinked hydrogel, and PVA-chitosan as a chemically crosslinked hydrogel.

As stated in Section 4, for each type of crosslinking, four different samples were investigated. Three samples with lecithin additions at different concentrations (0.5, 1, and 2 wt.%) were labeled according to their lecithin concentration (i.e., “0.5”, “1” and “2”). The fourth sample was a reference sample without lecithin, simply marked as “R”. The lecithin concentrations were selected on the basis of preliminary experiments focused mainly on estimating the maximum amount of lecithin that could be incorporated into the hydrogel matrix.

### 2.1. Physical Crosslinking

Agarose was a representative of the physically crosslinked hydrogel matrix, whose properties were affected by lecithin content. Hydrogel samples after preparation as well as samples after the drying and rehydration procedure were studied (schematic figure of the preparation procedure can be seen in the Supplementary Materials Figure S1).

#### 2.1.1. Rheology

**Figure 1:**
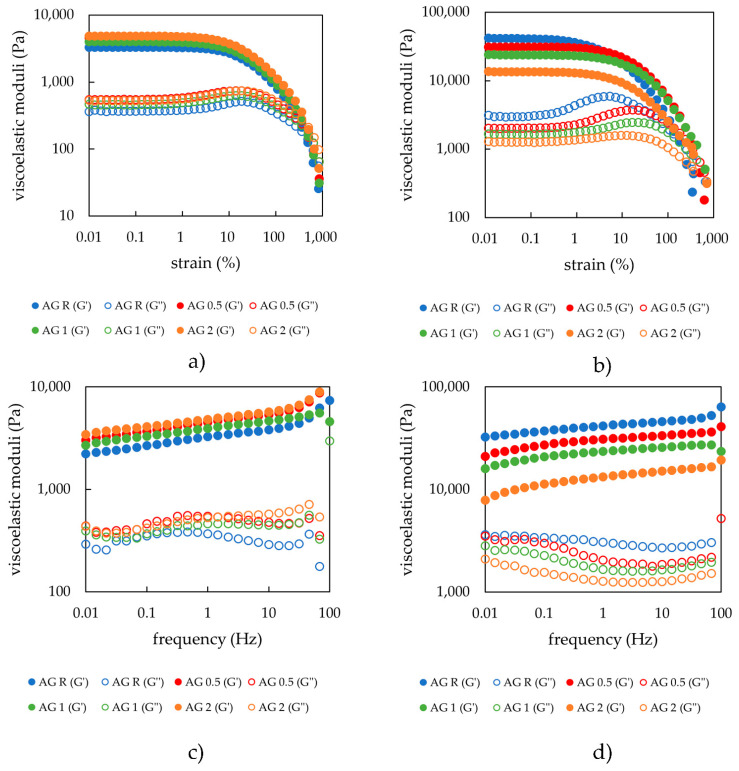
(**a**) Strain sweep of agarose hydrogels with different lecithin concentrations (0, 0.5, 1, and 2 wt.%) after preparation; (**b**) strain sweep of agarose hydrogels with different lecithin concentrations (0, 0.5, 1, and 2 wt.%) after drying and rehydration of the xerogels; (**c**) frequency sweep of agarose hydrogels with different lecithin concentrations (0, 0.5, 1, and 2 wt.%) after preparation; (**d**) frequency sweep of agarose hydrogels with different lecithin concentrations (0, 0.5, 1, and 2 wt.%) after drying and rehydration of the xerogels.

Amplitude sweep results for physically crosslinked hydrogels obtained under an applied oscillatory strain of 1 Hz suggest that differences in lecithin concentration have, from a viscoelastic property point of view, a minimal influence on the hydrogel structure after preparation, especially with respect to the width of the linear viscoelastic region (as can be seen in Figure 1a). The storage as well as the loss modulus gradually increased with increasing lecithin concentration, which might be due to the overall higher dry content of the hydrogels. The effect of lecithin concentration on the viscoelastic properties of agarose hydrogels was also minimal in the linear viscoelastic region (LVR), which is the range of the values of storage modulus where the hydrogel is able to resist the applied oscillatory strain and can thus indicate the strength of non-covalent hydrogel nodes. Probably, the strength of the physically crosslinked hydrogel is provided mainly by non-covalent weak interactions (H-bonding) between the chains of agarose. Lecithin only had a small effect on the viscoelastic properties of 1 wt.% aq. agarose. The obtained values marking the end of the LVR were very similar for all samples physically crosslinked (Table 1). The values reported in the tables were either obtained by rheology software (TRIOS TA Instruments) analyses (cross-over point, average moduli values in LVR) or calculated. The end of LVR was obtained by comparing the average value of storage modulus in LVR with each point, where the deviation greater than 5% marked the end of the LVR. The mesh size calculations are described in Section 4.2. The cross-over point (G′ = G″), the point at which the hydrogel was irreversibly damaged, was very similar for all samples.

The same amplitude sweep tests were performed on samples dried to the xerogel form and again rehydrated. The amount of absorbed water had a significant effect on these samples. As can be seen from Figure 1b as well as from the dry matter content experiments (Section 2.1.2), the samples with the highest lecithin content were able to reabsorb the largest amount of water (twice as much water as the sample without lecithin). This was also reflected in the amplitude sweep results because the moduli values for these hydrogels decreased proportionately. The reference sample had the highest moduli values, whereas the lowest values were observed for the samples with the greatest lecithin concentrations. The moduli values were somewhat larger than those for the samples studied after preparation (Table 2), mainly due to the elevated values of the swelling degrees of the systems after drying and rehydration in comparison with those of the just prepared hydrogels. Lecithin, therefore, favored water absorption. For the physically crosslinked hydrogels, even the cross-over point was affected, and samples with higher lecithin concentrations shifted the cross-over point to higher strain values. This could be the effect of the attractive interactions between lecithin and the polysaccharide chains, leading to the reinforcement of the hydrogels obtained after their drying and rehydration. In the initially prepared hydrogels, lecithin was dispersed to a greater extent in a liquid medium without this (strong) effect. This could be explained by the H-bonding between polysaccharide chains and lecithin, which are more significant for the rehydrated hydrogels because of the absence of water (in xerogel), which could not interfere. The same could be observed for the cross-over point, which again gradually increased with lecithin concentration.

Frequency sweep test results are presented in Figure 1 and show that the shape of the rheograms for all hydrogel samples was very similar. The storage modulus was dominant, which means that the samples act as a fully crosslinked gel material with a fully crosslinked internal structure. The trend of the moduli values was the same as that observed for the amplitude sweep tests and therefore indicates that the lecithin addition increased the values of the storage moduli as well as of the loss moduli, which was well correlated with the higher dry matter content, as previously stated. With increasing oscillation frequency values, the moduli values increased, which means that the hydrogel samples were not completely relaxed, and the degree of relaxation was influenced by the type of crosslinking. Practically, the average relaxation time of the hydrogel network exceeds the period associated with the progressively increasing frequency of the applied oscillatory deformations. The values of the mesh size of the internal structure of the hydrogels calculated from the frequency sweep tests using Equations (3) and (4) are recorded in Table 1 and Table 2. The results for the freshly prepared hydrogels showed the same trend as other rheological data (i.e., that the mesh size does not differ substantially between the concentrations), whereas for the rehydrated xerogels, a slight increase could be observed at higher lecithin concentrations, which can be explained by lecithin fitting itself into the pores and thus increasing its size. This could be explained by lecithin forming lamellar liquid-crystalline structures in absorbed water along with the already mentioned H-bonding between the polysaccharide chains and lecithin. When comparing the absolute values of mesh sizes for freshly prepared and rehydrated hydrogels, we can see that the pores decreased in size after rehydration.

**Figure 2:**
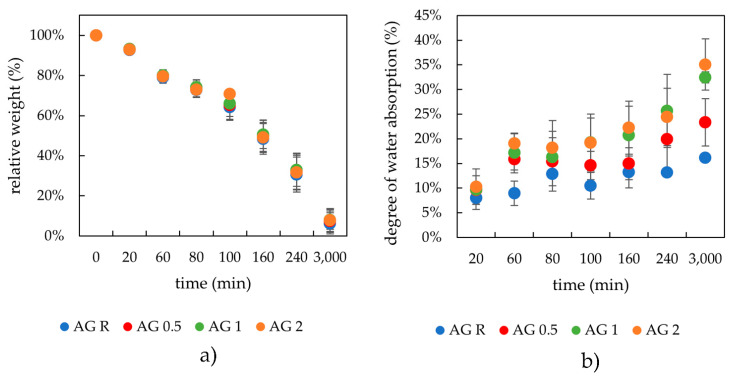
Drying (**a**) and rehydration (**b**) of the physically crosslinked agarose hydrogels with different contents of lecithin.

Based on the results of the strain and frequency sweeps performed onto the freshly prepared agarose hydrogels, it can be seen that lecithin, as an amphiphilic natural component, does not lead to a substantially modified viscoelastic behavior of these physically crosslinked hydrogels in the range of lecithin concentrations used (see Figure 1a,c). Agarose, which forms a thermoreversible physical hydrogel in an aqueous medium in the form of a natural linear polysaccharide, was not expected to interact significantly with amphiphilic lecithin. Thus, it was not expected that agarose could significantly interact with amphiphilic lecithin. Lecithin thus serves only as a filler, and does not interfere significantly with the internal structure of the hydrogel. Therefore, lecithin plays an important role in the rehydration of dried samples. Thus, an increasingly higher content of lecithin in the structure of such type of hydrogels causes the viscoelastic moduli storage and loss moduli to gradually decrease. Practically, the presence of lecithin affects the ability of agarose xerogels (hydrogel after drying) to reabsorb water (i.e., to swell) (see Figure 1b,d and Figure 2). The final viscoelastic properties of hydrogels are definitely affected by the amount of dispersion medium (water) after the swelling of xerogels. If the addition of lecithin, as the modifier of the internal architecture of hydrogels, is able to change the swelling properties, it will also definitely change the viscoelastic properties due to the different amount of water. From the applicative point of view, this finding is absolutely essential, given that by choosing a suitable concentration of additive (lecithin), we were able to prepare hydrogels with the required properties (especially viscoelastic) tailored to a specific purpose.

#### 2.1.2. Drying and Rehydration Measurements

The amounts of water and dry matter associated with the studied gels are two of the most important parameters for hydrogel characterization and future applicability. Dry matter affects the behavior of the final material. The same is true for the water inside the hydrogel, which significantly affects, for example, the transport properties. As stated in Section 1, these parameters predetermine the applicative nature of the final system.

The results of the drying kinetics of physically crosslinked hydrogels can be seen in Figure 2a. At the start of these experiments, all weights of the hydrogels (2 ± 0.2 g) and xerogels were comparably the same. It can be seen that the lecithin addition had no influence on the drying kinetics. The most likely explanation is that water retained by lecithin is not bound as tightly as water hydrating agarose. Conversely, during the swelling process, hydrogel with lecithin easily draws water (more easily than the agarose hydrogel solely) and this resulted in the lecithin-agarose samples showing a higher swelling ability with corresponding lower moduli (Figure 1). The swelling experiments demonstrated the influence of lecithin on the swelling capacity. Therefore, the lecithin structures insert themselves into the hydrogel pores and support the water intake. The kinetics of the swelling process was very similar for all samples, with a peculiarity noted at the onset of the experiment, where the samples richer in lecithin (1 and 2 wt.%) revealed a greater rate of water absorption. Additionally, the same systems (agarose with 1 and 2 wt.% of lecithin) were able to absorb the largest amount of water.

#### 2.1.3. Morphological Characterization of Xerogels

**Figure 3:**
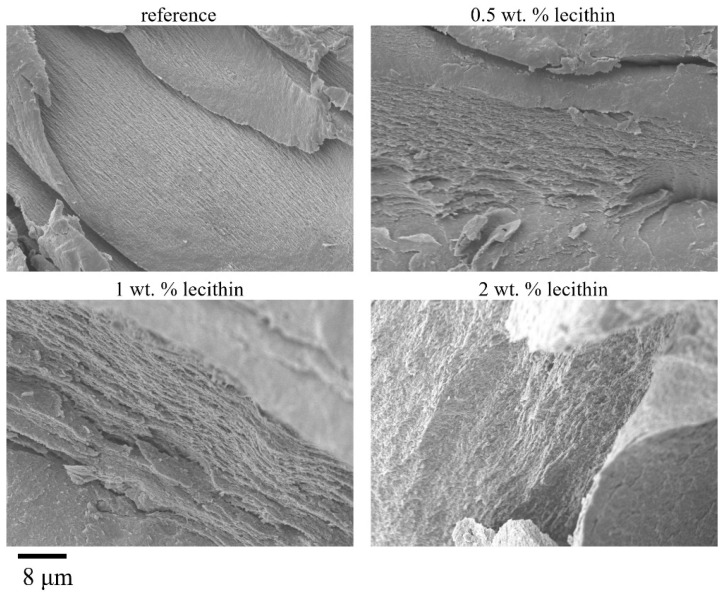
Physically crosslinked agarose xerogels with different lecithin contents observed in sectional view by SEM. Magnification 5000×.

Morphological characterization was performed on dried samples; therefore, the results may not correspond to the results obtained from methods where hydrogels are studied in native form (specifically, rheology). From the results obtained by scanning electron microscopy (SEM), the effect of lecithin addition could be observed in sectional view. The surfaces of these xerogels were smooth and with no visible pores on the micrometer scale. In sectional view, the lecithin-free xerogel exhibited a layered structure of polymer fibers with no visible interferences (see Figure 3). The same layered morphology was also observed for xerogels of agarose with different contents of lecithin even though there were regions of fusion of adjacent layers. Overall, the general morphology, practically devoid of pores as revealed by SEM, is most likely due to a compact structure resulting via the air drying procedure applied to hydrogels to finally obtain xerogels.

For these xerogels, gas sorption measurements were also performed (Table 3). The low values of the specific surface suggest a lack of the pore structure of xerogels, with a slight dependence on the compactness of layered morphology of these systems in dry state. Even if the results of gas sorption are in line with those of SEM investigation, the gas sorption method is not quite a suitable technique for determining the structure of these xerogels.

### 2.2. Ionic Crosslinking

Sodium alginate crosslinked by the calcium chloride in the two to one weight ratio was a representative of the ionically crosslinked hydrogel matrix, where the negatively charged poly(guluronic) acid units of alginate (-COO^−^) interact with the polyvalent ions (Ca^2+^) to form a bond (schematic figure of the preparation procedure can be seen in Supplementary Materials, Figure S2). The final properties were also affected by lecithin addition. Hydrogel samples, both after preparation and dried and rehydrated, were studied by rheology, drying, and rehydration as well as morphological characterization.

#### 2.2.1. Rheology

**Figure 4:**
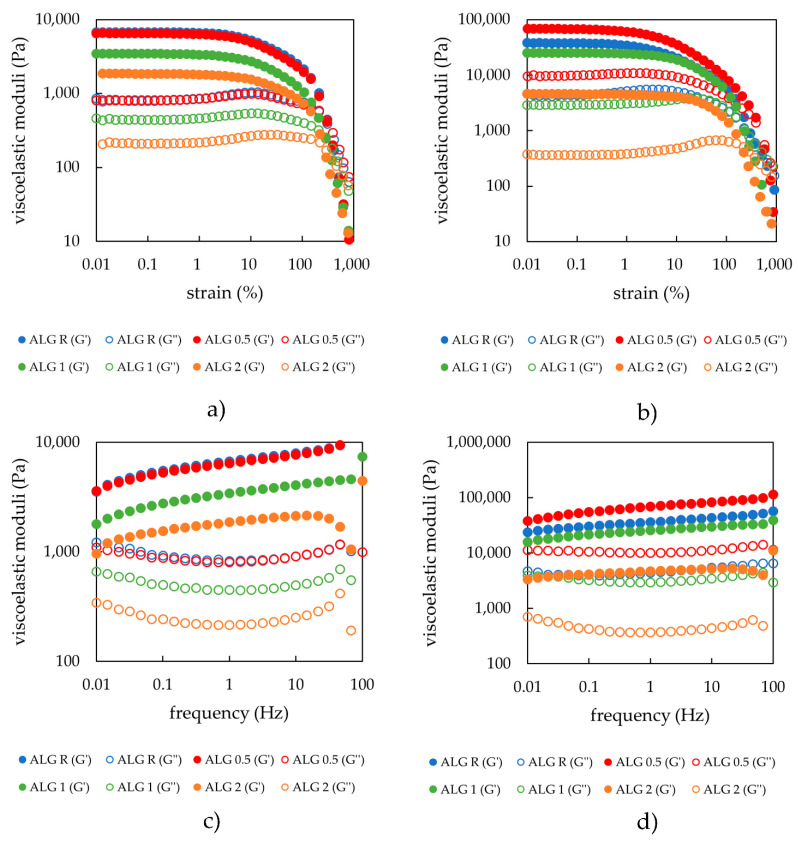
(**a**) Strain sweep of alginate hydrogels with the addition of different lecithin concentrations (0, 0.5, 1, and 2 wt.%) after preparation; (**b**) strain sweep of alginate hydrogels with different lecithin concentrations (0, 0.5, 1, and 2 wt.%) after drying and rehydration of the xerogels (frequency applied 1 Hz); (**c**) frequency sweep of alginate hydrogels with different lecithin concentrations (0, 0.5, 1, and 2 wt.%) after preparation; (**d**) frequency sweep of alginate hydrogels with different lecithin concentrations (0, 0.5, 1, and 2 wt.%) after drying and rehydration of the xerogels.

Ionically crosslinked hydrogels also underwent amplitude sweep tests. What is immediately observable is the decreasing trend of moduli for the freshly prepared samples as lecithin content increase (see Figure 4a). One of the reasons for this is the water intake during gelling, which increases for samples with ascending lecithin concentration (Section 2.2.2), the amphiphilic component playing a major role in the preparation of ionically crosslinked hydrogels. Larger lecithin addition also modified some characteristics of the hydrogels (see Table 4). The average moduli values in LVR steadily decreased after lecithin addition, thus making the gel softer. The most likely explanation is that after the crosslinking of alginate by calcium ions, free calcium chloride is still present in the system and is able to interact with the added lecithin micelles due to its dissociated form. Higher lecithin content causes a competitive interaction and as a result, lecithin displaces the calcium ions in the crosslinked alginate. Further lecithin could interact with the alginate via quaternary ammonia or with the calcium ions via negatively charged phosphate residues. For the moduli decrease, we could suggest that newly formed nodes are weaker and, in a lesser amount compared with the original alginate gel. Such competitive interactions were observable even during sample preparation, where the precipitate was visible on the surface of the solution. They were also confirmed by viscosity measurements, where the solution of calcium chloride and lecithin had higher viscosity values than expected, based on the viscosity of lecithin in water and of calcium chloride in water (figure is available in Supplementary Materials Figure S3). Other rheological data were very similar for the samples and, as stated earlier, the biggest differences were in the moduli values, thus in the hydrogel strength.

The rehydrated samples followed a similar trend with respect to the moduli values, where these values decreased with increasing lecithin concentration. Average moduli values in LVR reported in the table below (Table 5) were higher than those presented in Table 4 because the rehydrated samples were not able to reabsorb the same amount of water as the freshly prepared hydrogels. Such behavior could be due to a compact arrangement favored by non-covalent interactions (mainly ionic interactions induced by Ca^2+^ ions onto both alginate and lecithin components) during the drying process.

The rheograms obtained during the frequency sweep tests (expressed as viscoelastic moduli on applied frequency) (Figure 4c) obeyed the same order as those that resulted from the amplitude sweep tests (storage and loss moduli as a function of oscillatory applied strain of 1 Hz) (Figure 4a) for all the studied alginate and alginate-lecithin hydrogels. The calculated mesh size from the rheological (frequency sweep) measurement for freshly prepared ionically crosslinked alginate hydrogels indicated the effect of lecithin on the structural properties of these hydrogels. The higher addition of lecithin causes a higher mesh size (more than 50% if the hydrogels without/with 2 wt.% of lecithin is compared). The effect of lecithin concentration was also not observed for dried and rehydrated hydrogels. Although ionically crosslinked hydrogels have the ability to reabsorb the dispersion medium and again create a network internal structure by water intake, the internal structure of these hydrogels is probably damaged by the air-drying process. Moreover, swelled hydrogels differ in mesh size values in comparison with freshly prepared (e.g., hydrogels with 2 wt.% of lecithin had a mesh size of 17.3 nm while the mesh size of the hydrogels with the same concentration of lecithin after swelling was 7.6 nm). Therefore, the effect of lecithin on the mesh size of hydrogels repeatedly prepared by drying and swelling in water medium was negligible.

#### 2.2.2. Drying and Rehydration Measurements

**Figure 5:**
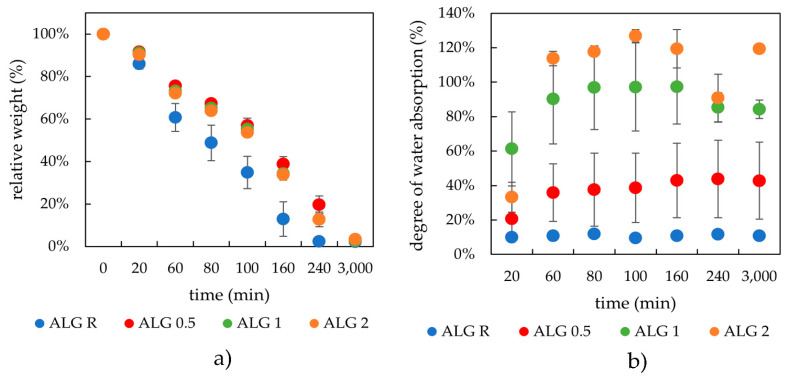
Drying (**a**) and rehydration (**b**) of ionically crosslinked alginate hydrogels with different lecithin content.

The drying curves for the alginate-lecithin systems were very similar almost irrespective of the lecithin content, in contrast to the drying dependence obtained for the freshly prepared hydrogels of alginate solely (Figure 5a). The different kinetics regarding the rate of water loss during the drying step could be due to the way lecithin fills the hydrogel pores and holds water within, and also due to the favorable electrostatic Ca^2+^-lecithin interactions, which influence the hydrogel structure and thus enable it to better hold water. As for the swelling after drying, it can be observed that the samples with higher lecithin concentrations were able to absorb water more rapidly and to a higher capacity, which is again due to the modified hydrogel network due to the presence of lecithin.

#### 2.2.3. Morphological Characterization of Xerogels

**Figure 6:**
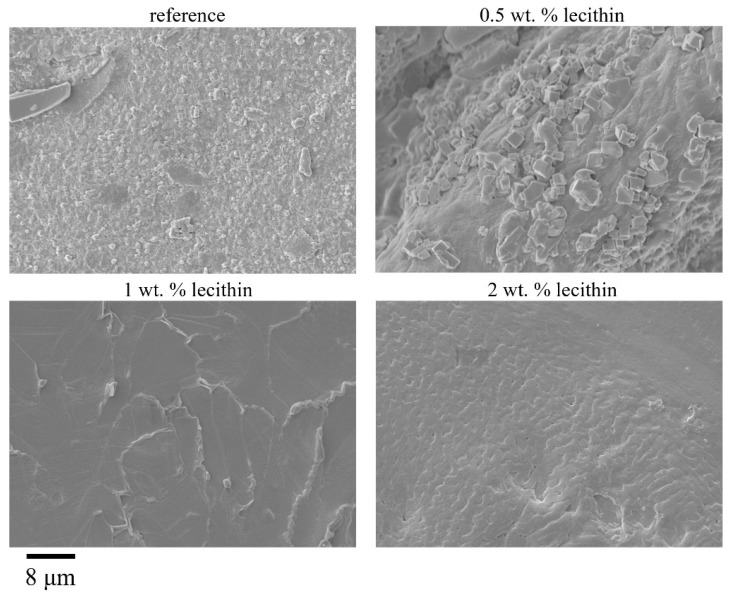
Surface morphologies of ionically crosslinked alginate xerogels with the addition of lecithin revealed by SEM. Magnification 5000×.

SEM images taken for xerogels prepared by ionic crosslinking show the effect of lecithin on the surface morphology of the samples (see Figure 6). Surface morphology of lecithin-free samples and of those with 0.5 wt.% lecithin exhibited a roughness due to the many micrometer-sized crystals of CaCl_2_ resulted after air-drying. Instead, the surface of xerogels with 1 and 2 wt.% lecithin is practically devoid of crystalline aggregates, with some degree of roughness, which led to a more compact structure of these mixed systems in their dry state. The morphological characteristics microscopically revealed are in accordance with the decreasing tendency of the specific surface values (from gas sorption measurements, Table 6) as the lecithin content rose. On the other hand, the lack of CaCl_2_ crystalline aggregates for the systems with a higher lecithin content (1 and 2 wt.%) could be related to Ca^2+^ consumption in favorable electrostatic interactions with lecithin anions, which means that the crystalline structures observed in the case of alginate xerogels without lecithin and for those with 0.5 wt.% lecithin could be due to the excess of CaCl_2_ contained in these explored samples. 

### 2.3. Chemical Crosslinking

Poly(vinyl alcohol) and chitosan crosslinked by the epichlorohydrin was a representative of the chemically crosslinked hydrogel matrix. Epichlorohydrin reacts with either the hydroxyl group of PVA or amino group of chitosan to form a highly reactive intermediate. This intermediate product reacts with another hydroxyl (PVA) or amino group (chitosan) to form the crosslinked structure. Study of these hydrogels, both in their freshly prepared state, after air-drying at 40 °C and their subsequent rehydration and as xerogels, showed some physico-mechanical properties altered by the lecithin content (schematic figure of the preparation procedure can be seen in the Supplementary Materials Figure S4).

#### 2.3.1. Rheology

**Figure 7:**
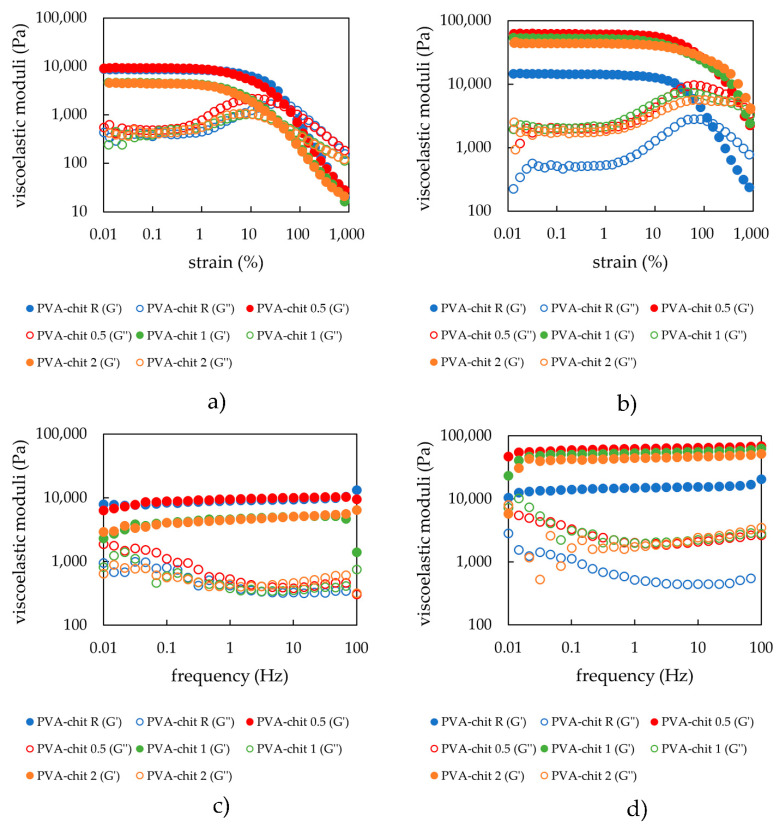
(**a**) Strain sweep of PVA-chitosan hydrogels with the different lecithin concentrations (0, 0.5, 1, and 2 wt.%) after preparation; (**b**) strain sweep of PVA-chitosan hydrogels with different lecithin concentrations (0, 0.5, 1, and 2 wt.%) after drying and rehydration of the xerogels (frequency applied–1 Hz); (**c**) frequency sweep of PVA-chitosan hydrogels with different lecithin concentrations (0, 0.5, 1, and 2 wt.%) after preparation; (**d**) frequency sweep of PVA-chitosan hydrogels with different lecithin concentrations (0, 0.5, 1, and 2 wt.%) after drying and rehydration of the xerogels.

For chemically crosslinked hydrogels, the amplitude sweep results showed that the addition of lecithin modified the rheological properties of hydrogels (see Figure 7a). However, the highest lecithin concentration did not lead to further changes in the mechanical properties. The same can be said after comparing the data points (see Table 7). At the same time, a higher content of lecithin decreased the values marking the end of the LVR as well as the strength of the hydrogels and the cross- over point values. The results are acceptable after taking into account the preparation and final state of the hydrogel. An important step of the preparation procedure is drying of the liquid mixture, which leads to crosslinking of the nodes and its subsequent rehydration. If lecithin is present, the rehydration is improved.

The same experiments were performed for hydrogel samples dried and rehydrated. The dried and rehydrated hydrogels with lecithin assembled into the pores ended up with modified properties (see Figure 7b), specifically, an increase in moduli values and a decrease in the values marking the cross-over point, in contrast to the reference sample. As can be seen in Figure 7b and Table 8, the presence of lecithin makes the hydrogels obtained after the drying–rehydration step much more deformation resistant, characterized by much higher values of strain at the cross-over point. At the same time, for these mixed rehydrated hydrogels, lecithin, irrespective of its content, exerted a larger influence in the enhancement of the hydrogels’ strength (average moduli values in LVR) when compared to the rehydrated systems physically and ionically crosslinked. 

The frequency and amplitude sweep results indicated the same tendency discussed above (see comparatively Figure 7). Thus, a critical lecithin concentration is necessary to modify the properties of this type of chemically crosslinked hydrogels (according to the results lying between 0.5 and 1 wt.%); also, there is a maximum concentration above which further modifications do not occur (differences between 1 and 2 wt.% are negligible). The significant difference in the chemically crosslinked hydrogels (comparing to the physically and ionically crosslinked) is the relaxation phenomenon characterized by much longer relaxation times in contrast to covalently crosslinked systems. Covalently crosslinked hydrogels exhibit almost constant values of storage moduli over the whole range of the applied frequencies. The same trend was also observed for the dried and rehydrated samples. Again, for all samples, the storage modulus prevailed in comparison to the loss modulus. The mesh sizes of these samples (Table 7 and Table 8) were not affected by the content of lecithin, a result that can be explained by the character of covalent crosslinking, which is stronger than physical and ionic crosslinking. On the other hand, the same trend of decreasing mesh sizes after rehydration could be observed.

#### 2.3.2. Drying and Rehydration Measurements

**Figure 8:**
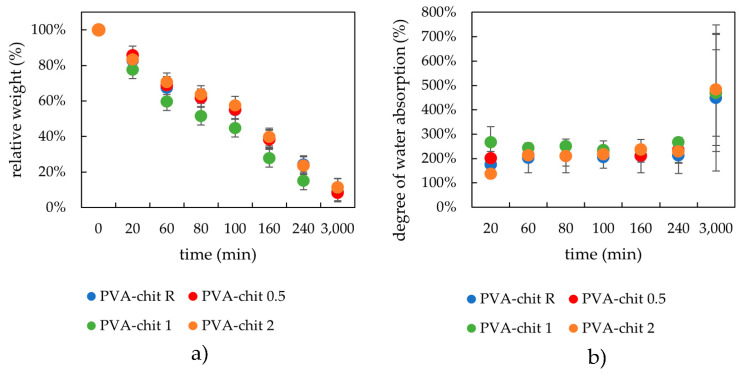
Drying (**a**) and rehydration (**b**) of chemically crosslinked PVA-chitosan hydrogels with different lecithin content.

As can be seen from Figure 8, the drying and swelling kinetics were not significantly altered by the addition of lecithin. Only a marginal influence was observed for samples with the highest lecithin concentrations, which were able to absorb the most water. This generally smaller influence of lecithin can be explained by the structure of chemically crosslinked hydrogels, which are characterized by a high enough crosslinking density and, consequently, by a smaller pore size morphology. The structure is more organized due to the stronger covalent bonds. The water absorption for this kind of hydrogel possessing stronger covalent cross linkages was very fast and occurred almost immediately during the first minutes of the swelling experiments.

#### 2.3.3. Morphological Characterization of Xerogels

**Figure 9:**
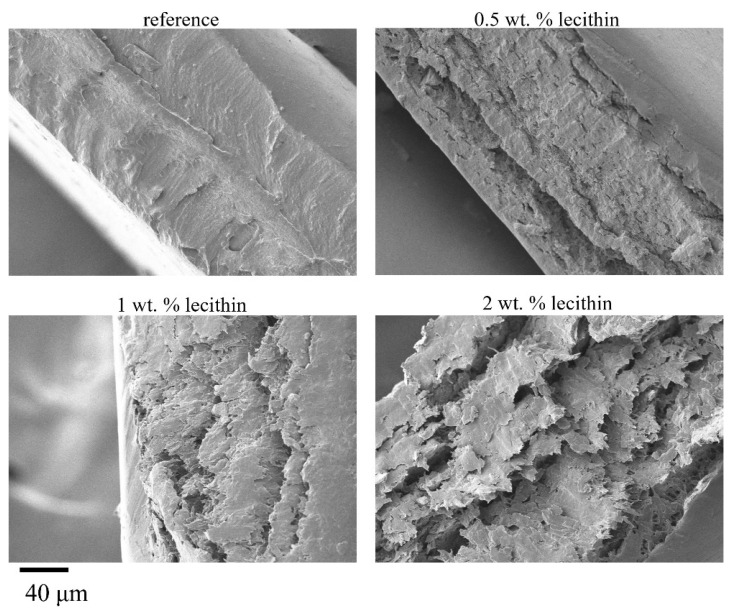
SEM images of chemically crosslinked PVA-chitosan xerogels with the addition of lecithin taken from the sectional view. Magnification 1000×.

Results on the structural characterization of chemically crosslinked xerogels were similar to those for physically crosslinked hydrogels. The surface morphology of these xerogels looked smooth with no visible pores. In sectional view, SEM images revealed clear layered structures, with an interlayer roughness increasing with lecithin content (Figure 9), which in turn led to a gradual ascension of the value of specific surface (Table 9). Despite this fact, an apparently less corrugated surface observed for lecithin-free hydrogels had a higher specific surface area (Table 9), which might be explained by a greater compactness associated with the layered structure of the mixed xerogels. 

## 3. Conclusions

This work studied the influence of lecithin (L-α-phosphatidylcholine) on three differently crosslinked hydrogels (physically crosslinked agarose, alginate ionically crosslinked by calcium ions, and a mixture of PVA and chitosan chemically crosslinked by epichlorohydrin). The bulk of this work was to study differences between the gels investigated immediately after preparation and the corresponding rehydrated xerogels (prepared by swelling). By choosing the lecithin content, we were able to modify some of the mechanical properties of the hydrogels with a modified internal structure, especially in the case of the rehydrated ones. In this regard, the addition of lecithin had the strongest influence in enhancing the strength of chemically crosslinked PVA-chitosan gels, which is partially consistent with the mesh size and by the amount of water absorbed into their structure after being previous air-dried. Apart from the rheological data and those obtained from the kinetics of water loss during hydrogel dehydration, these conclusions were supported by the scanning electron microscopy and gas sorption experiments performed on the xerogels. For this type of material, even though gas sorption appears to be inappropriate, however, it serves to confirm the non-porous structure of the xerogels.

In this work, we determined that the addition of phospholipid lecithin into the hydrogel matrix can alter their mechanical properties, which might be highly beneficial knowledge for the use of such hydrogels in particular applications. However, the transport properties also need to be investigated. Therefore, further transport experiments are required, which are absolutely crucial for a better understanding of such hydrogel materials and how they can be used in final applications.

## 4. Materials and Methods

Hydrogels with distinct gelation mechanisms (physical, ionic, chemical crosslinking) [20] were studied. As an example of a physically crosslinked matrix, the linear thermoreversible polysaccharide agarose (Agarose E, Condalab, Madrid, Spain) at 1 wt.%, was used [21]. As an example of an ionically crosslinked matrix, sodium alginate (Sigma-Aldrich, Prague, Czech Republic) at 2 wt.% crosslinked by calcium chloride (Lach-Ner, Neratovice, Czech Republic) at a two to one weight ratio was chosen [22]. For chemically crosslinked hydrogels, poly(vinyl alcohol) (Sigma-Aldrich, Prague, Czech Republic) mixed with chitosan (low molecular weight, Sigma-Aldrich, Prague, Czech Republic) and crosslinked by epichlorohydrin (Sigma-Aldrich, Prague, Czech Republic) was employed [23]. L-α-Phosphatidylcholine (lecithin) was incorporated into all hydrogel samples before gelation at three different weight percentage concentrations (Sigma-Aldrich, Czech Republic, Prague).

The materials and their concentrations and ratios were selected on the basis of data previously reported [20,21,22,23,24] and can be seen in the table below (Table 10).

### 4.1. Water Loss during Drying and Rehydration Measurements

The ability to hold, release, and absorb water was tested by different approaches. Water loss was monitored by means of simple drying tests. All samples were dried either in the laboratory dryer at 40 °C and regularly weighed, or in a semi-automatic moisture analyzer (IR-35, Denver Instrument, Denver, CO, USA), where the weight was recorded automatically. The relative weight of the hydrogel (*x*) during drying was calculated using the following formula:

(1) $$x=\frac{m_{t}}{m_{0}}\cdot 100$$

where *m*_t_ is the weight of the gel at time t, and *m*_0_ is the weight of the hydrogel in the swollen state.

Often very small weight losses of water from the hydrogel samples made using drying scales more difficult. For this reason, drying kinetics were mostly studied using the combination of laboratory driers and analytical scales, upon which samples were weighed every twenty minutes. After the samples were dried to the xerogel form, they were inserted into a water bath, where they were kept until they reached their maximum water absorption capacity. The degree of water absorption (*m*_a_) was calculated by:

(2) $$m_{a}=\frac{m_{t}}{m_{x}}\cdot 100$$

where *m*_t_ is the weight of the hydrogel at time t, and *m*_x_ is the weight of the xerogel. The hydrogel samples were regularly weighed on analytical scales to study their swelling kinetics.

### 4.2. Rheology

Hydrogels are semi-solid materials that exhibit distinctive mechanical characteristics lying between those of solids and liquids. Therefore, rheology is indeed an appropriate technique for studying their behavior [25,26,27,28,29]. The mechanical properties of the prepared hydrogels were determined by rheological characterization using a rotational rheometer (Discovery HR-2, TA Instruments) employing cross-hatched 20 mm plate–plate geometry to avoid potential sensor wall-slippage during measurement. The complex rheological procedure consisted of strain sweep and frequency sweep tests. The strain sweep test is a useful tool for obtaining information about samples if fluid-like or gel-like behavior under different values of applied strain prevails. In addition, it is possible to determine the region where the deformation is non-destructive (the linear viscoelastic region-LVR) as well as the behavior of the sample when the LVR strain limit is exceeded. The other mentioned test, the frequency sweep test, serves the purpose of describing hydrogel behavior in the non-deformation range (LVR) and provides information about different crosslinking sites (if applicable) in the internal structure of the hydrogel. Both tests were carried out on freshly prepared samples and rehydrated ones. The rehydrated samples were first dried to constant mass in the laboratory dryer for two days at a constant temperature of 40 °C and further rehydrated for three days in distilled water. Freshly prepared agarose and alginate samples were measured within a gap of 1000 µm. The gap for rehydrated samples varied according to the thickness of the gel, which depended on its swelling capacity, 500 µm for agarose gels and 1000 µm for alginate gels. PVA-chitosan hydrogels (both fresh and rehydrated) were measured within a gap of 200 µm due to the limited thickness of the prepared hydrogel foils. Prior to each applied test, samples were allowed to temper and rest for 180 s after loading into the measuring gap. 

To obtain a suitable value of constant amplitude strain for the linear viscoelastic region (LVR), which was an essential parameter for ongoing frequency sweep tests, strain sweep tests were conducted first within the amplitude strain range of 0.01–1000% under a constant frequency of oscillation of 1 Hz in at least two repetitions, using a freshly loaded sample for each test. From these measurements, a strain of 0.1% was chosen as a suitable value of deformation for ongoing frequency tests, because this strain value lays within the LVR for all fresh and rehydrated samples. The range of oscillating frequencies for the frequency sweep tests was set to 0.01–100 Hz. Like the former strain sweep tests, the frequency sweep tests were also conducted in at least two repetitions. A summary of settings for both rheology tests is presented in Table 11. 

Routine techniques that are usable for the characterization of the internal structures of many materials (e.g., scanning electron microscopy) have some limitations in the study of hydrogels. One of the most limiting factors is that the structures of hydrogels are mostly studied in a dried state. The internal structures of a hydrogel in the presence of water and in the absence of water must certainly differ. Moreover, the preparation of the hydrogel in its dried state is also critical because the dispersion medium (water) must be removed (mostly by evaporation or by sublimation if lyophilization is used). Unfortunately, both of these processes (evaporation as well as sublimation) have a significant impact on the final xerogel morphology. Simply, the fragile internal structure of the hydrogel may be critically damaged by the removal of the dispersion medium. Thus, such a resulting structure (specifically, the porous structure) revealed by scanning electron microscopy often has low informative value with respect to the internal structure of the hydrogel in its swollen state. Therefore, an alternative way to determine the pore size (and then obtain information about the internal structure of the hydrogel) must be found. An interesting solution to this problem is offered by the rheological characterization of the hydrogel, which involves the calculation of the mesh size.

Mesh size, as one of the most critical parameters in hydrogel characterization, was calculated by means of relaxation spectra (relaxation moduli *G* and relaxation time *λ*) from the frequency sweep oscillation measurements in accordance with the Maxwell model [30]. The frequency sweep (viscoelastic moduli as a function of oscillation frequency) was interpolated by continuous relaxation spectra in TRIOS software (TA Instruments, New Castle, DE, USA). 

Typical relaxation spectra can be found in the Supplementary Materials (Figure S5). On the basis of previous rheological investigation [25], it was concluded that the optimal number of Maxwell elements was 4, in order to fit the frequency sweep measurements of the hydrogels. Four relaxation moduli were obtained from continuous relaxation spectra analyses. The sum of relaxation moduli was calculated in order to determine the crosslinking density [31] (see Equation (3), where *ρ*_x_ represents the crosslinking density (mol·m^−3^)) and provides information on the density of the junction in the swollen hydrogel form. *G* (Pa) is the sum of 4 relaxation moduli, *R* (J·mol^−1^·K^−1^) represents the universal gas constant, and *T* is the thermodynamic temperature in Kelvins.

(3) $$\rho_{x}=\frac{G}{RT}$$

If all criteria are met (in particular, frequency sweep measurements are realized in the linear viscoelastic region and the mechanical properties of hydrogels with different crosslinking are consistent with rubber elasticity theory [32]), finally the mesh size can be calculated using Equation (4), where *ξ* is the mesh size (unit: m) and N_A_ represents Avogadro’s number.

(4) $$\xi =\sqrt[{3}]{\frac{6}{\pi \rho_{x}N_{A}}}$$

### 4.3. Morphological Characterization of Xerogels

Since the structure affects properties that are crucial for hydrogel applications, determining the hydrogel morphology is one of the most important characterizations. There are many direct (microscopy) and indirect (scattering-based) methods to characterize hydrogel morphology [33]. Several direct visualization techniques (light microscopy, laser scanning confocal microscopy, and micro-computed tomography) that can handle swollen hydrogels have considerable disadvantages (e.g., limited resolution) [34]. On the other hand, commonly used scanning electron microscopy includes a critical step (i.e., the inevitable solidification of the sample using drying or freezing, during which the collapse of the structure or the creation of artifacts can occur) [35,36]. Kaberova et al. [37] tested the usability of scanning electron microscopy and concluded that the results from this method should always be confirmed by microscopy techniques applicable for gels in their swollen state.

For the characterization of dry samples, the specific surface area (the Brunauer–Emmett–Teller (BET) approach) is typically determined. The specific surface area is not suitable for characterizing hydrogels because of the already mentioned artifacts that appear during the preparation of dried samples. However, it can be used, for example, for the characterization of materials used in a dried state and that can form hydrogels (adsorbent) [38], or for the confirmation of reversible porosity [39].

The structure of the xerogels was studied in this work. Specifically, scanning electron microscopy and gas sorption were chosen as suitable techniques for determining the internal architecture of xerogels. Since the mechanical properties were studied for hydrogels right after preparation and also for swollen hydrogels after dehydration, it seemed convenient to investigate the structural properties of the hydrogels in these forms. Since this form is a dry form, it was possible to avoid deformation of the structure caused by the preparation of hydrogels for scanning electron microscopy.

#### 4.3.1. Scanning Electron Microscopy

To determine changes in hydrogel structure, xerogels of all prepared samples were subjected to direct visualization using scanning electron microscopy. The samples were dried in a laboratory dryer at 40 °C. A few small specimens were taken from each studied sample to maintain objective observation. These specimens were subsequently gold-coated in a sputtering device (POLARON) and investigated using a ZEISS EVO LS 10 scanning electron microscope.

Both the surface morphologies and sectional images of samples were recorded. Observations were realized in secondary electron (SE) mode and the accelerating voltage was set to 5 kV to avoid charging of the samples.

#### 4.3.2. Gas Sorption

A NOVA 2200e high-speed gas sorption analyzer (Quantachrome Instruments) was used to determine the specific surface area. The samples were weighed into a measuring cell (0.05–0.1 g). The measuring cell was placed in a degassing station, where the degassing process was carried out at 75 °C for 20 h. After cooling, the degassed sample was weighed to four decimal places. The samples were placed in a measuring station. The adsorption and desorption isotherms were measured under liquid nitrogen (77 K) from 0.05–0.95 of the relative pressure P/P_0_. The obtained data were processed by NovaWin software and specific surface area was calculated by the multi-point BET method.

## Figures and Tables

**Table 1:** Values for physically crosslinked agarose hydrogels after preparation obtained from strain and frequency sweep tests before drying.

	Cross-Over Point		Average Moduli Values in LVR		End of LVR	Mesh Size
Lecithin Concentration	G′	Strain	G′	G″	Strain	Mesh
(wt.%)	(Pa)	(%)	(Pa)	(Pa)	(%)	(nm)
0 (R)	157.5 ± 4.1	425.8 ± 2.2	3299 ± 277	366 ± 28	2.5 ± 1.0	13.3 ± 0.1
0.5	207.9 ± 2.1	414.1 ± 4.5	4576 ± 12	551 ± 15	1.8 ± 0.0	13.4 ± 0.4
1	194.9 ± 10.7	433.2 ± 10.2	4002 ± 81	461 ± 4	1.8 ± 0.0	12.7 ± 0.1
2	224.5 ± 0.0	468.0 ± 2.9	4880 ± 27	529 ± 8	1.8 ± 0.0	12.9 ± 0.3

**Table 2:** Values obtained from strain and frequency sweep tests for physically crosslinked agarose hydrogels after drying and rehydration.

	Cross-Over Point		Average Moduli Values in LVR		End of LVR	Mesh Size
Lecithin Concentration	G′	Strain	G′	G″	Strain	Mesh
(wt.%)	(Pa)	(%)	(Pa)	(Pa)	(%)	(nm)
0 (R)	1814 ± 340.6	250.4 ± 131.7	41,386 ± 10,517	2977 ± 707	0.3 ± 0.1	7.6 ± 1.1
0.5	1005.7 ± 142.9	718.5 ± 129.3	31,216 ± 980	2010 ± 4	1.2 ± 0.5	7.6 ± 0.2
1	542.7 ± 0.0	1148.7 ± 0.0	23,829 ± 3	1642 ± 118	1.4 ± 0.3	8.2 ± 0.1
2	350.8 ± 35.5	1257.6 ± 12.2	13,506 ± 1217	1256 ± 122	0.9 ± 0.0	9.0 ± 0.3

**Table 3:** Specific surface area for physically crosslinked agarose xerogels with the addition of lecithin determined by gas sorption.

Concentration of Lecithin (wt.%)	Specific Surface Area (m^2^/g)
0 (R)	3.4
0.5	1.0
1	1.9
2	2.1

**Table 4:** Values for ionically crosslinked alginate hydrogels after preparation obtained from strain and frequency sweep tests before drying.

	Cross-Over Point		Average Moduli Values in LVR		End of LVR	Mesh Size
Lecithin Concentration	G′	Strain	G′	G″	Strain	Mesh
(wt.%)	(Pa)	(%)	(Pa)	(Pa)	(%)	(nm)
0 (R)	150.4 ± 9.1	260.4 ± 18.6	1667 ± 192	165 ± 23	1.7 ± 0.0	10.9 ± 0.4
0.5	158.6 ± 12.5	275.3 ± 24.4	2138 ± 480	245 ± 66	1.3 ± 0.0	11.0 ± 0.7
1	110.8 ± 1.2	260.8 ± 5.4	1052 ± 1	104 ± 0	1.6 ± 0.3	13.8 ± 1.9
2	65.3 ± 17.9	278.2 ± 9.0	468 ± 15	41 ± 0	2.1 ± 0.4	17.3 ± 1.5

**Table 5:** Values for ionically crosslinked alginate hydrogels after drying and rehydration obtained from the strain and frequency sweep tests.

	Cross-Over Point		Average Moduli Values in LVR		End of LVR	Mesh Size
Lecithin Concentration	G′	Strain	G′	G″	Strain	Mesh
(wt.%)	(Pa)	(%)	(Pa)	(Pa)	(%)	(nm)
0 (R)	479.2 ± 129.7	210.8 ± 119.7	26,342 ± 13,355	3191 ± 1346	1.6 ± 0.4	4.6 ± 1.4
0.5	894.9 ± 612.4	522.6 ± 51.8	68,513 ± 17,434	9861 ± 1533	0.6 ± 0.6	12.3 ± 2.1
1	1179.5 ± 106.7	209.1 ± 37.3	25,386 ± 741	2912 ± 45	1.2 ± 0.2	8.3 ± 1.8
2	553.5 ± 24.3	189.5 ± 17.4	4599 ± 500	1842 ± 1447	2.4 ± 0.0	7.6 ± 0.5

**Table 6:** Specific surface area for ionically crosslinked alginate xerogels with the addition of lecithin determined by gas sorption.

Concentration of Lecithin (wt.%)	Specific Surface Area (m^2^/g)
0 (R)	9.1
0.5	6.3
1	5.9
2	4.7

**Table 7:** Values for chemically crosslinked PVA-chitosan hydrogels obtained from strain and frequency sweep tests before drying.

	Cross-Over Point		Average Moduli Values in LVR		End of LVR	Mesh Size
Lecithin Concentration	G′	Strain	G′	G″	Strain	Mesh
(wt.%)	(Pa)	(%)	(Pa)	(Pa)	(%)	(nm)
0 (R)	1665.3 ± 43.2	53.8 ± 8.2	8629 ± 304	398 ± 4	1.6 ± 0.3	13.6 ± 0.7
0.5	1005.5 ± 32.4	49.4 ± 18.4	6644 ± 1503	307 ± 44	1.2 ± 0.9	13.8 ± 0.6
1	666.6 ± 5.4	40.2 ± 3.2	4545 ± 129	377 ± 68	0.6 ± 0.1	12.7 ± 0.1
2	631.6 ± 24.7	39.1 ± 4.7	4398 ± 195	421 ± 5	0.7 ± 0.1	12.9 ± 0.1

**Table 8:** Values for chemically crosslinked PVA-chitosan hydrogels after drying and rehydration obtained from strain and frequency sweep tests.

	Cross-Over Point		Average Moduli Values in LVR		End of LVR	Mesh Size
Lecithin Concentration	G′	Strain	G′	G″	Strain	Mesh
(wt.%)	(Pa)	(%)	(Pa)	(Pa)	(%)	(nm)
0 (R)	2470.0 ± 494.7	138.5 ± 13.5	14,514 ± 1413	532 ± 33	3.2 ± 0.0	11.6 ± 0.3
0.5	7122.4 ± 633.3	379.1 ± 233.0	62,099 ± 6505	1928 ± 65	5.0 ± 1.0	7.1 ± 0.1
1	4964.6 ± 275.8	502.2 ± 277.5	52,833 ± 10,153	2089 ± 246	3.0 ± 1.7	6.2 ± 1.3
2	4074.2 ± 182.3	900.1 ± 97.5	43,685 ± 3177	1761 ± 211	5.9 ± 2.3	8.1 ± 0.0

**Table 9:** Specific surface area for chemically crosslinked PVA-chitosan xerogels with different lecithin content determined by gas sorption.

Concentration of Lecithin (wt.%)	Specific Surface Area (m^2^/g)
0 (R)	2.9
0.5	0.8
1	1.2
2	1.6

**Table 10:** Concentrations of each individual component in the final hydrogel form (agarose, sodium alginate, calcium chloride, PVA, chitosan, and lecithin).

**Physically Crosslinked Hydrogels**			
**Sample**	**Agarose (wt.%)**		**Lecithin (wt.%)**
AG R	1		0
AG 0.5	1		0.5
AG 1	1		1
AG 2	1		2
**Ionically Crosslinked Hydrogels**			
**Sample**	**Sodium** **Alginate (wt.%)**	**Calcium Chloride** **(mol·dm^3^)**	**Lecithin** **(wt.%)**
ALG R	2	0.1	0
ALG 0.5	2	0.1	0.5
ALG 1	2	0.1	1
ALG 2	2	0.1	2
**Chemically Crosslinked Hydrogels**			
**Sample**	**PVA (wt.%)**	**Chitosan (wt.%)**	**Lecithin** **(wt.%)**
PVA R	7.8	2.5	0
PVA 0.5	7.8	2.5	0.5
PVA 1	7.8	2.5	1
PVA 2	7.8	2.5	2

**Table 11:** Summary of settings for rheology measurements (conditioning step, amplitude sweep, and frequency sweep).

**Conditioning Step**			
Temperature		25 °C	
Time		180 s	
**Amplitude Sweep**		**Frequency Sweep**	
temperature	25 °C	temperature	25 °C
strain	0.01–1000%	strain	0.1%
points per decade	8	points per decade	6
frequency	1 Hz	frequency	0.01–100 Hz

## Data Availability

The data used in this study are available on request from the corresponding author.

## Supplementary Materials

The following supporting information can be downloaded at: www.mdpi.com/article/10.3390/gels8020115/s1, Figure S1: Preparation procedure of physically crosslinked agarose hydrogels; Figure S2: Preparation procedure of ionically crosslinked alginate hydrogels; Figure S3: Dynamic viscosity measurements for combinations of solutions of lecithin, CaCl_2_ and alginate; Figure S4: Preparation procedure of chemically crosslinked PVA-chitosan hydrogels; Figure S5: Typical relaxation spectra for mesh size calculations, TRIOS software (TA Instruments).
