# Supplementary material for: Novel Hydrogel Material with Tailored Internal Architecture Modified by “Bio” Amphiphilic Components—Design and Analysis by a Physico-Chemical Approach
Source: Gels. 2022 Feb 13;8(2):115. doi: 10.3390/gels8020115 (PMC8872166; doi:10.3390/gels8020115)
Supplement: Supplementary file 1 [file gels-08-00115-s001.zip › Supplementary_Materials_Heger_et_al_Gels_2022.pdf]

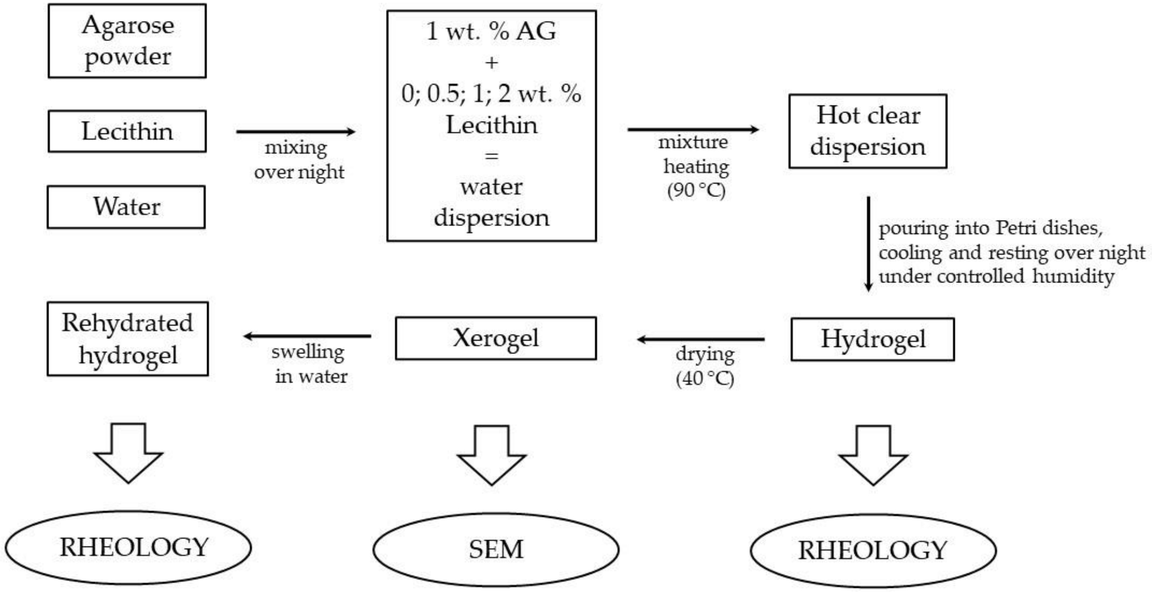

Figure S1. Preparation procedure of physically crosslinked agarose hydrogels.

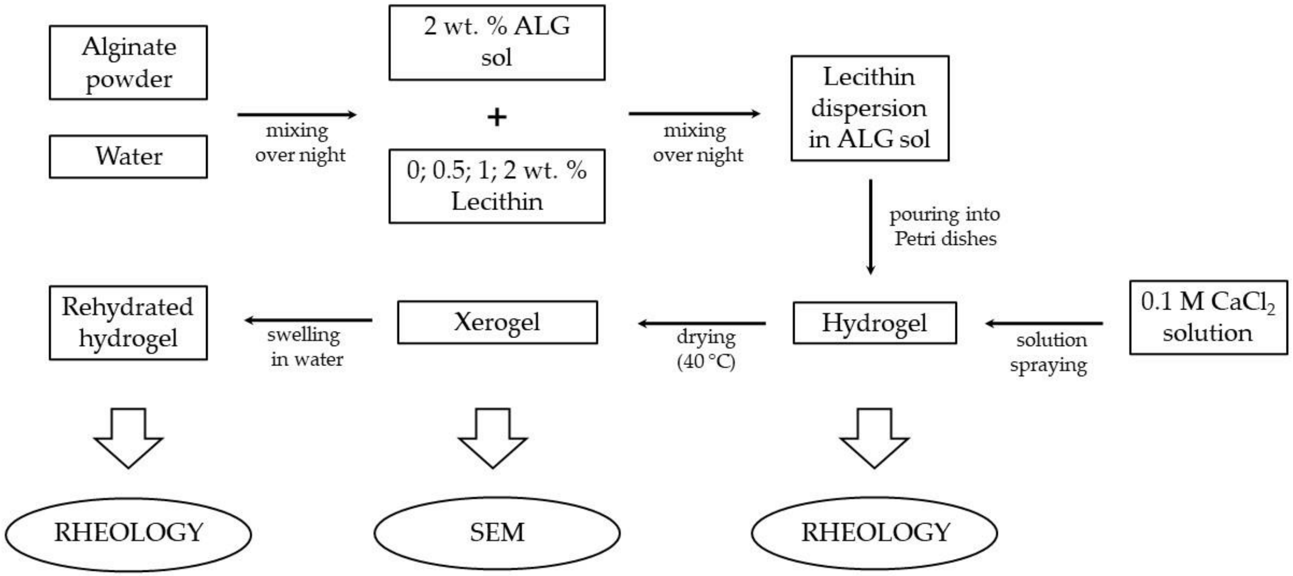

Figure S2. Preparation procedure of ionically crosslinked alginate hydrogels.

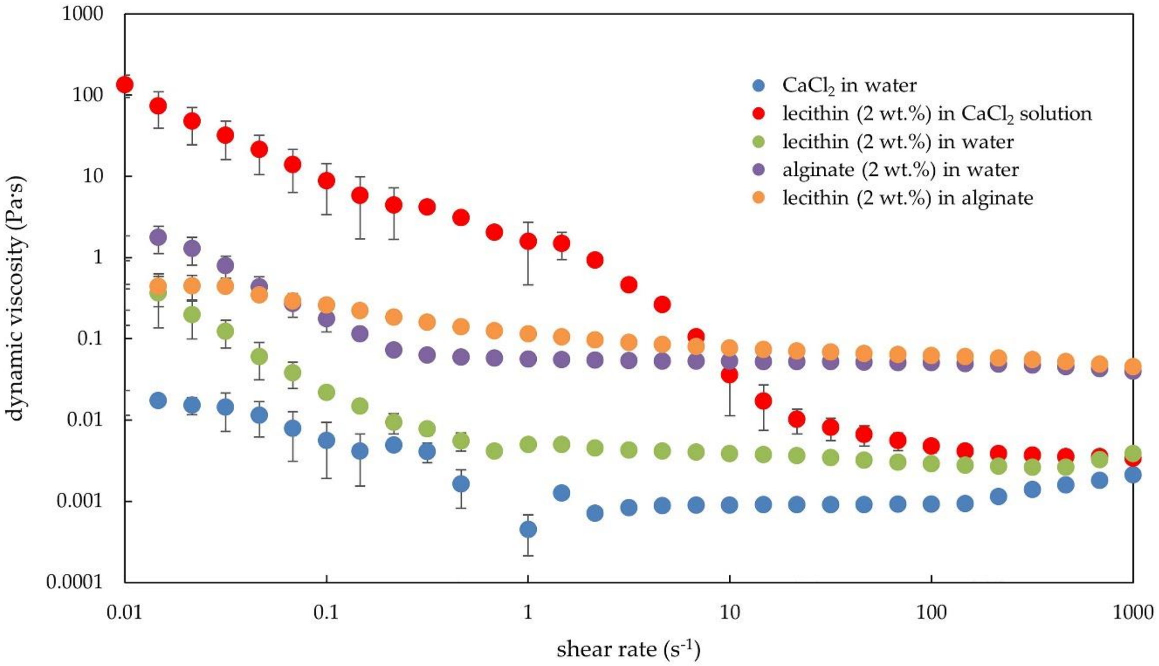

Figure S3. Dynamic viscosity measurements for combinations of solutions of lecithin, $CaCl_2$ and alginate.

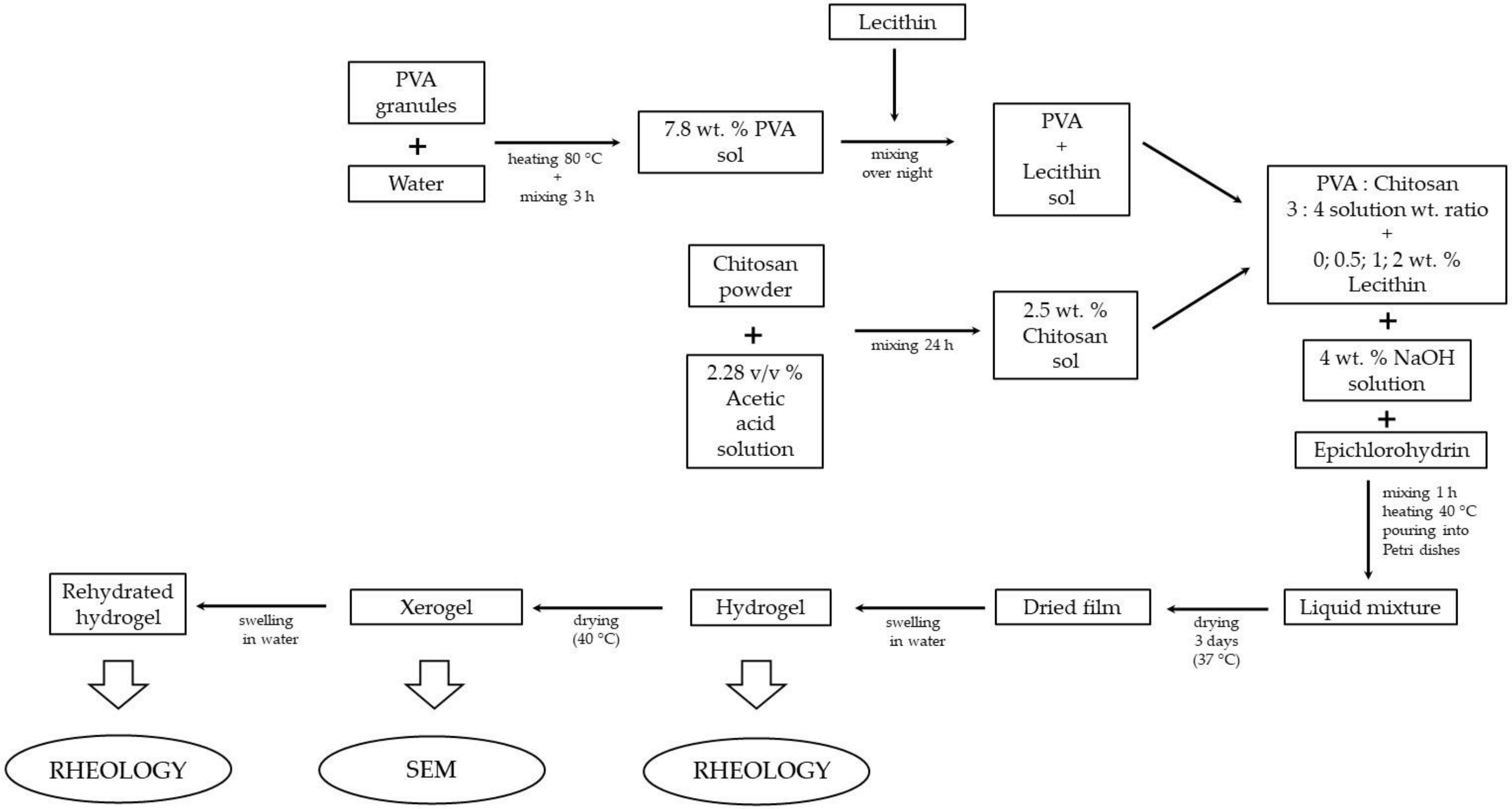

Figure S4. Preparation procedure of physically crosslinked PVA-chitosan hydrogels.

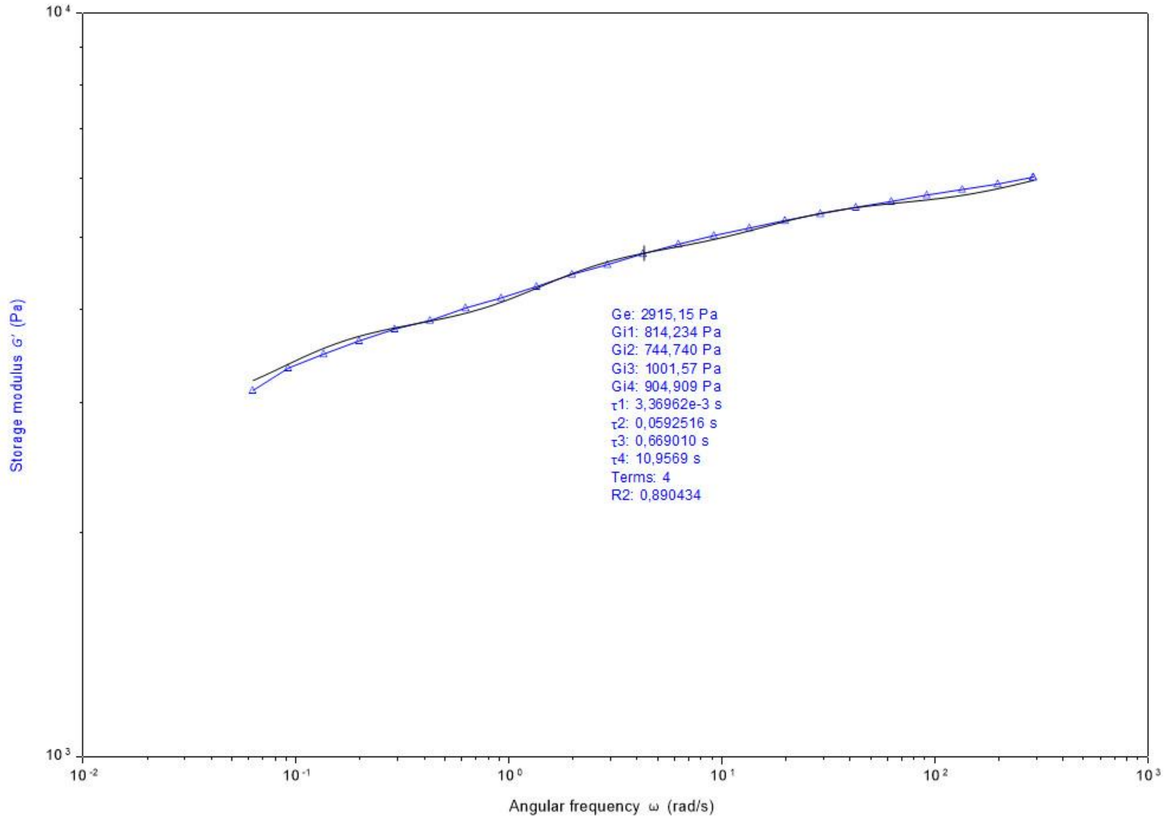

Figure S5. Typical relaxation spectra for mesh size calculations, TRIOS software (TA Instruments).
